# An interaction study of online learning satisfaction with parent-child relationships and trait coping styles

**DOI:** 10.3389/fpsyt.2024.1320886

**Published:** 2024-03-01

**Authors:** Leji Wen, Weizhuo Xu, Xiaoyue Yan, Xin Lin, Chen Shao, Lei Zhang

**Affiliations:** ^1^ National Innovation Platform for Industry-Education Integration in Vaccine Research, School of Public Health, Xiamen University, Xiamen, Fujian, China; ^2^ Department of Urology, Xiang’an Hospital of Xiamen University, School of Medicine, Xiamen, Fujian, China

**Keywords:** online learning, parent-child relationships, trait coping styles, satisfaction, adolescents

## Abstract

As the physical and mental development of the young is not only influenced by the parent-child relationship (PR) and the student's academic performance, but also moderated by trait coping styles (TCS), the changes between these three during the online learning period in an epidemic need to be reconsidered. This study aims to explore the factors affecting online learning satisfaction (OLS) among students and their interaction with parent-child relationship and trait coping style. A web-based questionnaire was employed, encompassing general information, the Trait Coping Style Questionnaire (TCSQ), and queries related to OLS. A total of 1,287 valid questionnaires were collected, with 593 from junior high school students, 197 from high school students, and 497 from university students. Our findings indicate that parent-child relationship (PR), positive coping style (PCS), and learning status (LS) showed a positive correlation with OLS (*r*=0.110, *P*<0.001; *r*=0.786, *P*<0.001). Conversely, negative coping style (NCS) presented a negative correlation with OLS (*r*=-0.186, *P*<0.01). Multiple regression analysis of OLS reveals that PR has a significant impact on OLS (*P*<0.001, *β*=0.291), as does LS (*P*<0.001, *β*=0.767). However, trait coping styles (TCS) appear to have no significant effect on OLS. Notably, PR plays a significant and positive mediating role between LS and OLS, with a mediation effect of 0.0132 (*P*<0.05), accounting for 1.682% of the total effect. These findings suggest that strengthening parent-child interactions and fostering adaptive coping mechanisms could play a crucial role in enhancing students' satisfaction with online education. Such improvements could potentially contribute to superior academic outcomes and overall student well-being.

## 1 Introduction

The COVID-19 pandemic has disrupted the traditional education mode, leading many young individuals to explore alternatives through online platforms (1). E-learning, delivered via digital media, offers flexible and remote learning opportunities. Online learning has several advantages (2), such as optimizing information retention, enhancing accessibility, and increasing engagement. However, online learning during the pandemic has presented challenges (3), including the quality of interactions between students and teachers, course design, hardware and software availability and reliability, instructor support and guidance, as well as student motivation and self-regulation. All these factors influence online learner’s satisfaction and academic performance (4).

Learning Status (LS) refers to an individual’s comprehensive state regarding cognitive, emotional, motivational, physiological, and environmental conditions that influence their learning process and effectiveness. Learning Satisfaction pertains to students’ overall contentment and feelings towards their educational experience, including course content, teaching methods, resources, environment, and learning outcomes (5–7). It is a vital indicator of education quality and effectiveness, reflecting the active participation and intrinsic value that students experience during the learning process (8). Learning Status is frequently analyzed in conjunction with learning patterns, engagement, and attendance to support research efforts (9). And a study by Cavite (10) identified a significant positive correlation between learning engagement and satisfaction in online learning.

The Parent-Child Relationship (PR) refers to the socio-emotional bond between parents and children, typically encompassing love, trust, communication, guidance, and support (11, 12). This relationship is fundamental to the family structure and significantly impacts an individual’s development and socialization process (13).

Current research in this field is notably diverse. A key area of interest is the influence of parental behavior or socioeconomic status on their offspring. For instance, a review of parental involvement and academic performance revealed that increased parental engagement and elevated expectations are correlated with enhanced student performance (14). Additionally, the dynamics of parent-child interactions, encompassing communication and cooperation, are under scrutiny. Research has established that discordant parent-child relationships can negatively affect the child’s mental health, potentially leading to serious conditions like depression (15). Furthermore, learning satisfaction is recognized as a critical determinant of academic success, underscoring the importance of continued investigation in this area (16).

Extensive research on the effects of parent-child relationships on satisfaction with learning in traditional classrooms show that harmonious parent-child relationships can boost adolescents’ concentration levels, enhance teacher-student communication (17), and increase learning efficiency. These positive outcomes contribute to an increased satisfaction among adolescents with traditional classroom learning (14). In contrast, the effect of parent-child relationship on online learning satisfaction remains under-explored. Therefore, in this study, we aim to investigate factors affecting online learning satisfaction and to explore the influence of the parent-child relationship on Online Learning Satisfaction (OLS).

Coping serves a crucial mediator in the psychological stress process, influencing both the nature and eventual outcome of stressful events (18). “Trait Coping Styles” (TCS) denote the stable elements of coping that are associated with personality traits and mental health (19). The Trait Coping Style Questionnaire (TCSQ) is a self-report scale designed to assess individuals’ coping strategies in response to life challenges. It delineates positive and negative attitudes and responses to adversity, offering insights into the steadiness of coping strategies (19). Predominantly used in mental health studies, the TCSQ has been applied to investigate the correlation between coping styles and anxiety and depression in adolescents (20), as well as the moderating effect of coping styles on perceived stress of COVID-19 patients (21). A cross-sectional study of university students during the COVID-19 epidemic highlighted the considerable role of coping plays in E-learning satisfaction (22), with findings indicating that Positive Coping Styles (PCS) can mitigate the adverse effects of study-related stress and environmental changes (23).

During the COVID-19 pandemic, factors like learners’ current learning status and the design of online courses have been crucial in determining Online Learning Satisfaction (OLS). The implementation of quarantine policies resulted in less outdoor activity, increased physical proximity, and enhanced shared time between parents and children, potentially influencing the parent-child relationship dynamics and, consequently, the children’s educational and personal lives (24). Research has underscored the importance of addressing parent-adolescent relationships during the COVID-19 pandemic, particularly for adolescents dealing with high stress levels and engaging in active coping strategies. For those with lower active coping levels, parental support might compensate for the lack of peer interaction (25). Given the importance of the parent-child relationship as a predictor of academic performance, understanding its impact on OLS is essential. Investigating the theoretical model linking the parent-child relationship, learning status, and OLS is thus of paramount importance for enhancing the efficacy of online learning for student populations. Additionally, incorporating the TCSQ into our survey facilitates an examination of how positive and negative coping styles influence online learning satisfaction and provides a measure of the questionnaire’s overall reliability.

## 2 Materials and methods

### 2.1 Object and method

According to Kendall’s criterion for sample size (1975), the number of observations should be at least tenfold the number of variables. Considering a 20% expected rate of non-recoverable questionnaires due to invalid responses, the minimum sample size was determined to be 270 cases. This figure is based on multiplying the sum of items from the general demographic survey (n=4), questions related to Online Learning Satisfaction (OLS) (n=2), Parent-child Relationship (PR) (n=1), and Trait Coping Style Questionnaire (TCSQ) (n=20) by 10. Through a combination of convenience and snowball sampling techniques, 1297 questionnaires were initially collected. Post-quality review, 1287 of these questionnaires were considered valid, resulting in a valid response rate of 99.23%.

### 2.2 Tool

#### 2.2.1 Self-developed general information questionnaire

The survey collected basic personal information, such as gender, grade, place of residence, and health status. It also included questions on the following topics: Parent-child relationship: Has your relationship with your parents changed since the outbreak? (Single choice, Score: 1: Very bad-5: Very good); Health status (Ibid); Learning status (LS): Are you satisfied with your learning status during online learning? (Single choice, Score: 1: Very dissatisfied-5: Very satisfied); Online Learning Satisfaction: What is your overall evaluation of online learning? (Ibid).Note: “Ibid” refers to the use of the same scoring scale (1 to 5) for health status, learning status, and online learning satisfaction.

#### 2.2.2 Trait Coping Style Questionnaire

The questionnaire consists of 20 statements accompanied by a scale ranging from 1 to 5. It includes 10 statements related to positive coping (PC) and 10 statements related to negative coping (NC). The respective scores for PC or NC were obtained by summing the relevant item scores, with higher scores indicating more pronounced positive or negative coping characteristics. To evaluate the reliability of the scale, Cronbach’s alpha was employed. Given that some question items on the TCSQ scale were negatively correlated with the total scale, the internal consistency of the data requiring reverse scoring was evaluated using Cronbach’s alpha coefficient. The raw alpha coefficient was 0.82, and Guttman’s lambda6 coefficient was 0.85, indicating good internal consistency of the data.

#### 2.2.3 Quality control

To ensure the accuracy and reliability of the questionnaire outcomes, participants were given detailed explanations regarding the survey’s objectives, its importance, and necessary precautions. In the online questionnaire, all items were designated as required to eliminate the incidence of incomplete data. Furthermore, to address any privacy issues, the questionnaires were disseminated anonymously. Quality control measures on the 1287 collected responses included the exclusion of entries with a response time under 120 seconds. For the 41 students unsure about their residency status, their responses were proportionally assigned to an alternate residence category through random allocation.

#### 2.2.4 Statistical analysis

SPSS 24.0 was used for data cleaning, scale reliability assessment, descriptive statistics for data frequencies and percentages, and normality tests for measurements. We used histograms, P-P plots, or Q-Q plots for graphical observation as our preferred method for normality testing. In cases where graphical interpretations were ambiguous, the Kolmogorov-Smirnov and Shapiro-Wilk tests were applied. For data conforming to a normal distribution with chi-square homogeneity of variance, two independent samples t-tests and ANOVAs were utilized. non-normal distributions warranted the use of Mann-Whitney U-tests or Kruskal-Wallis H-tests. Logistic regression analyses were conducted to explore correlations. To examine the moderated mediation effects of Parent-child Relationships and Trait Coping Styles on Online Learning Satisfaction, the bootstrapping method in SPSS PROCESS macro (version 3.5, Model 4) was implemented. The test level was set at α=0.05.

## 3 Results

### 3.1 Demographic characteristics of students

The study surveyed a total of 1,287 participants (**Table 1**), with a predominance of female participants, who constituted 55.56% (n=715), while males accounted for 44.44% (n=572). Nearly half of the participants, 47.55% (n=612), hailed from rural localities. The demographic breakdown revealed that junior high school students formed the largest segment at 46.08% (n=593), university students comprised 38.62% (n=497), and high school students represented 15.31% (n=197). Statistically significant disparities were observed in the participant demographics concerning their place of residence and gender (χ² = 809.824, *P*< 0.001; χ² = 9.541,*P*< 0.01), reflecting diversity within the study sample.

**Table 1:** Demographic characteristics.

		Education			n/N	χ²	*P*
		high	middle	college			
Gender	Female	302(50.90%)	117(59.40%)	296(59.60%)	715(55.56%)	9.541	0.008
	Male	291(49.10%)	80(40.60%)	201(40.40%)	572(44.44%)		
Residence	directly governed	4(0.70%)	35(17.80%)	25(5.00%)	64(4.97%)	809.824	<0.001
	Provincial capital	9(1.50%)	34(17.30%)	114(22.90%)	157(12.20%)		
	prefecture-level	21(3.50%)	101(51.30%)	183(36.80%)	305(23.70%)		
	County-level	39(6.60%)	15(7.60%)	95(19.10%)	149(11.58%)		
	Townships and Rural	520(87.70%)	12(6.10%)	80(16.10%)	612(47.55%)		
n/N		593(46.08%)	197(15.31%)	497 (38.62%)	N=1287		

### 3.2 The analysis of TCS

Analysis of the TCS questionnaire scores (**Table 2**) revealed that student groups varied in negative coping (NC) and positive coping (PC). Specifically, university students recorded the highest PC scores (33.75), whereas high school students registered the highest NC scores (30.15) (*H*=9.624, *P*<0.01; *H*=22.087, *P*<0.001) (*P*<0.05) (**Table 2**). Moreover, female students demonstrated higher NC scores than male students (30.17 vs. 28.518, *Z*=-3.690, *P*<0.001), with NC scores also varying based on the place of residence (*H*=9.624, *P*<0.01). Further subgroup analysis (**Table 3**) indicated that female junior high school students scored slightly lower in PC than their male counterparts (*Z*=-1.662, *P*<0.05). These findings suggests that students’ attitudes and behaviors in response to adversity—whether positive or negative—are influenced by their gender, geographic background, and educational level.

**Table 2:** Scores on the questionnaire.

	Item	N=1287	HS				PR			LS			OLS			PC			NC		
			X±S/M (P25, P75)	*Z*/*H*		*P*	X±S/M (P25, P75)	*Z*/*H*	*P*	X±S/M (P25, P75)	*Z*/*H*	*P*	X±S/M (P25, P75)	*Z*/*H*	*P*	X±S/M (P25, P75)	*Z*/*H*	*P*	X±S/M (P25, P75)	*Z*/*H*	*P*
**Education**	**Junior High School**	593	2.36±0.85**/**2(2,3)	2.941		0.230	3.27±0.73**/**3(3,4)	**14.657**	**0.001**	3.27±0.82**/**3(3,4)	**11.634**	**0.003**	3.44±0.88**/**3(3,4)	**18.378**	**<0.001**	33.36±5.22**/**33(30,36)	**22.087**	**<0.001**	28.71±7.84**/**30(24,34)	**9.624**	**0.008**
	**High School**	197	2.45±0.82**/**3(2,3)				3.09±0.58**/**3(3,3)			3.05±1.02**/**3(3,4)			3.19±0.85**/**3(3,4)			31.89±6.09**/**31(30,35)			30.15±6.89**/**30(27,33)		
	**University**	497	2.40±0.84**/**2(2,3)				3.23±0.63**/**3(3,3)			3.13±0.94**/**3(3,4)			3.25±0.94**/**3(3,4)			33.73±5.87**/**34(30,37)			29.99±6.68**/**30(26,34)		
**Gender**	**Female**	715	2.40±0.79**/**2(2,3)	**0.716**		**0.002**	3.21±0.61**/**3(3,3)	1.360	0.174	3.16±0.86**/**3(3,4)	0.853	0.393	3.33±0.87**/**3(3,4)	0.290	0.772	33.05±5.15**/**33(30,36)	-1.345	0.179	30.17±6.85**/**30(26,34)	**-3.690**	**<0.001**
	**Male**	572	2.37±0.91**/**2(2,3)				3.25±0.74**/**3(3,4)			3.21±0.96**/**3(3,4)			3.34±0.94**/**3(3,4)			33.55±6.20**/**33(30,37)			28.51±7.72**/**30(24,33)		
**Residence**	**directly governed**	64	2.53±0.87**/**3(2,3)	3.999		0.406	3.13±0.60**/**3(3,3)	1.448	0.863	3.19±0.97**/**3(3,4)	4.929	0.295	3.27±0.88**/**3(3,4)	**11.098**	**0.025**	31.84±6.03**/**31(29,36.5)	5.829	0.213	30.91±7.87**/**31(28,35.5)	**11.381**	**0.023**
	**Provincial capital**	157	2.44±0.8**/**3(2,3)				3.18±0.66**/**3(3,3)			3.11±0.94**/**3(3,4)			3.18±0.97**/**3(3,4)			33.87±5.84**/**34(30,37)			30.01±6.79**/**30(27,34)		
	**prefecture-level**	305	2.38±0.83**/**2(2,3)				3.25±0.63**/**3(3,3)			3.11±0.99**/**3(3,4)			3.24±0.95**/**3(3,4)			33.05±6.11**/**33(30,37)			30.03±6.76**/**30(26,34)		
	**County-level**	149	2.42±0.91**/**2(2,3)				3.23±0.70**/**3(3,3)			3.19±0.93**/**3(3,4)			3.38±0.9**/**3(3,4)			33.35±5.91**/**33(30,37)			29.8±7.36**/**30(26,35)		
	**Townships and Rural**	612	2.36±0.84**/**2(2,3)				3.24±0.70**/**3(3,4)			3.23±0.83**/**3(3,4)			3.41±0.86**/**3(3,4)			33.37±5.21**/**33(30,36.5)			28.74±7.55**/**30(24,34)		

The bold font denotes statistically significant p-value.

**Table 3:** Scores on the questionnaire for different levels of education.

		Item	n(N=1287)	HS			PR			LS			OLS			PC			NC		
				X±S/M (P25, P75)	*Z*/*H*	*P*	X±S/M (P25, P75)	*Z*/*H*	*P*	X±S/M (P25, P75)	*Z*/*H*	*P*	X±S/M (P25, P75)	*Z*/*H*	*P*	X±S/M (P25, P75)	*Z*/*H*	*P*	X±S/M (P25, P75)	*Z*/*H*	*P*
**Junior High School**	**Gender**	**Female**	302	2.36±0.78/2(2,3)	**0.244**	**0.016**	3.27±0.66/3(3,4)	**-0.226**	**0.001**	3.27±0.74/3(3,4)	**0.154**	**0.023**	3.47±0.82/3(3,4)	0.849	1.22	33.01±4.79/33(30,36)	**-1.662**	**0.013**	29.95±7.54/30(25,35)	3.972	0.356
		**Male**	291	2.35±0.93/2(2,3)			3.28±0.80/3(3,4)			3.26±0.89/3(3,4)			3.41±0.93/3(3,4)			33.72±5.62/33(30,37)			27.43±7.95/29(22,32)		
	**Residence**	**directly governed**	4	2.25±0.96/2.5(1.5,3)	2.513	0.642	3±1.41/3.5(2,4)	2.297	0.681	2.75±1.26/3(2,3.5)	3.608	0.462	2.50±1.00/3(2,3)	5.938	0.204	29.75±5.5/31(26.5,33)	5.49	0.241	31.25±16.46/31(18,44.5)	2.357	0.670
		**Provincial capital**	9	2.67±0.50/3(2,3)			3.44±0.73/3(3,4)			3.00±0.87/3(3,3)			3.11±1.17/3(3,4)			34.33±4.74/35(31,36)			30.00±8.08/32(22,37)		
		**prefecture-level**	21	2.48±0.81/2(2,3)			3.38±0.50/3(3,4)			3.05±0.97/3(3,3)			3.33±0.97/3(3,4)			34.24±5.35/34(31,38)			27.81±6.85/28(26,31)		
		**County-level**	39	2.41±0.99/3(2,3)			3.38±0.94/3(3,4)			3.38±0.88/3(3,4)			3.56±1.10/4(3,4)			31.54±5.81/32(28,35)			30.03±7.99/30(26,35)		
		**Townships and Rural**	520	2.34±0.85/2(2,3)			3.26±0.72/3(3,4)			3.28±0.8/3(3,4)			3.45±0.85/3(3,4)			33.47±5.16/33(30,37)			28.61±7.8/30(24,34)		
**High School**	**Gender**	**Female**	117	2.44±0.80/3(2,3)	-0.046	0.507	3.06±0.51/3(3,3)	-0.776	0.076	2.97±0.99/3(3,4)	-1.192	0.378	3.16±0.83/3(3,4)	-0.606	0.448	31.91±5.23/31(29,35)	0.049	0.053	30.21±5.97/30(28,34)	0.13	0.055
		**Male**	80	2.45±0.86/3(2,3)			3.13±0.66/3(3,3)			3.15±1.06/3(3,4)			3.24±0.89/3(3,3.5)			31.86±7.21/31(30,36)			30.08±8.09/30(26.5,33)		
	**Residence**	**directly governed**	35	2.49±0.95/2(2,3)	3.189	0.527	3.00±0.54/3(3,3)	1.310	0.860	3.37±1.00/3(3,4)	**9.616**	**0.047**	3.46±0.89/3(3,4)	6.173	0.152	30.83±6.6/30(28,36)	3.196	0.526	30.69±6.91/30(28,33)	2.739	0.602
		**Provincial capital**	34	2.53±0.71/3(2,3)			3.09±0.62/3(3,3)			3.00±0.85/3(3,3)			3.03±0.72/3(3,3)			32.50±5.46/32(30,36)			29.91±5.67/30(27,33)		
		**prefecture-level**	101	2.35±0.81/2(2,3)			3.12±0.60/3(3,3)			3.07±1.01/3(3,4)			3.18±0.89/3(3,4)			31.85±6.29/31(30,35)			29.65±7.57/30(27,34)		
		**County-level**	15	2.73±0.88/3(2,3)			3.00±0.53/3(3,3)			2.60±1.12/3(2,3)			3.20±0.56/3(3,3)			32.93±4.28/33(31,36)			32.93±6.10/32(27,37)		
		**Townships and Rural**	12	2.58±0.79/3(2,3)			3.17±0.39/3(3,3)			2.58±1.16/2.5(2,3)			3.00±1.04/3(2.5,3.5)			32.25±6.9/30(28.5,34)			30.00±4.47/30(28,32.5)		
**University**	**Gender**	**Female**	296	2.43±0.79/2(2,3)	0.749	0.111	3.20±0.59/3(3,3)	-0.978	0.250	3.12±0.90/3(3,4)	-0.359	0.157	3.24±0.91/3(3,4)	-0.395	0.529	33.56±5.41/34(30,37)	-0.806	0.117	30.37±6.44/30(26,35)	1.517	0.766
		**Male**	201	2.37±0.90/2(2,3)			3.26±0.67/3(3,4)			3.15±1.00/3(3,4)			3.27±0.97/3(3,4)			33.99±6.49/34(30,38)			29.44±7.01/30(26,34)		
	**Residence**	**directly governed**	25	2.64±0.76/3(2,3)	3.572	0.467	3.32±0.48/3(3,4)	7.446	0.114	3.00±0.87/3(3,3)	2.201	0.699	3.12±0.78/3(3,3)	2.178	0.703	33.60±4.96/34(29,38)	3.028	0.553	31.16±7.79/32(30,36)	4.809	0.307
		**Provincial capital**	114	2.39±0.84/2(2,3)			3.19±0.66/3(3,3)			3.16±0.97/3(3,4)			3.24±1.02/3(3,4)			34.25±6.01/34(30,38)			30.04±7.04/30(27,34)		
		**prefecture-level**	183	2.38±0.85/2(2,3)			3.30±0.66/3(3,4)			3.14±0.99/3(2,4)			3.27±0.98/3(3,4)			33.57±6.03/34(30,37)			30.49±6.23/30(26,34)		
		**County-level**	95	2.37±0.88/2(2,3)			3.20±0.59/3(3,3)			3.20±0.88/3(3,4)			3.34±0.85/3(3,4)			34.16±6.05/34(31,38)			29.21±7.21/30(24,34)		
		**Townships and Rural**	80	2.42±0.81/2(2,3)			3.10±0.56/3(3,3)			3.04±0.91/3(3,3)			3.19±0.86/3(3,4)			32.91±5.33/33(30,36)			29.38±6.15/30(26,34)		

The bold font denotes statistically significant p-value.

### 3.3 The analysis of learning status and satisfaction

Based on the scores for self-evaluation of learning state and satisfaction with online learning (**Table 2**), it is apparent that different student groups have varying assessments of their own learning status and levels of satisfaction with online learning (*H*=11.634, *P*<0.01: *H*=18.378, *P*<0.001). Specifically, junior high school students are more satisfied with their own learning status and express higher satisfaction with online learning. Moreover, students from different residential areas also show differing levels of satisfaction with online learning (*H*=11.098, *P*<0.05). Interestingly, students from economically less developed areas have higher OLS scores, which might be attributed to relaxed supervision by teachers and reduced interaction between home and school, thus leading to students enjoying more leisure time during online learning. Further analysis (**Table 3**) reveals that junior high school girls perform better than boys in terms of their learning status (*Z*=0.154, *P*<0.05). Additionally, high school students from different residential areas show varying learning statuses (*H*=9.616, *P*<0.05). In economically less developed areas, the absence of a conducive online learning environment at home may be the cause for this disparity, with urban high school students significantly outperforming their rural counterparts (3.37 vs. 2.58; *H*=9.616, *P*<0.05).

### 3.4 The analysis of parent-child relationships

During the period of online learning, different student groups have reported varying perceptions of their harmony with their parents (*H*=14.657, *P*<0.05) (**Table 2**). High school students, compared to junior high and college students, tend to perceive less harmony in their interactions with parents. This could potentially be related to the deepening generational gap during adolescence or the pressures associated with academic work (**Table 2**). Further analysis (**Table 3**) reveals that both male and female junior high school students generally enjoy harmonious relationships with their parents. However, boys have reported a slightly better relationship with their parents (*Z*=-0.226, *P*<0.001), which might be influenced by family values or status within the family.

### 3.5 The correlational analysis of variables

The correlation analysis (**Table 4**) found that, OLS was negatively correlated with health status (*r*=-0.138, *P*<0.001) and NC (*r*=-0.170, *P*<0.001), positively correlated with PR(*r*=0.239, *P*<0.001), LS (*r*=0.786, *P*<0.001), and PC (*r*=0.111, *P*<0.001); PR showed a positive correlation with health status (*r*=-0.157, *P*<0.001), a positive correlation with LS (*r*=0.236, *P*<0.001), a positive correlation with PC (*r*=0.263, *P*<0.001), and a negative correlation with NC (*r*=-0.147, *P*<0.001).

**Table 4:** The correlational analysis of variables.

item	1	2	3	4	5	6	7	8
1 Education	1							
2 Gender	-0.028	1						
3Residence	-0.085 ^**^	0.088 ^**^	1					
4Health Status	-0.006	-0.020	-0.043	1				
5 PR	0.045	0.033	0.031	-0.157^***^	1			
6 LS	0.002	0.026	0.049	-0.132^***^	0.236^***^	1		
7 OLS	-0.014	0.007	0.090 ^**^	-0.138^***^	0.239^***^	0.786^***^	1	
8 PC	0.099^***^	0.044	0.023	-0.174^***^	0.263^***^	0.110^***^	0.111^***^	1
9 NC	0.021	-0.113^***^	-0.090 ^**^	0.300^***^	-0.147^***^	-0.186^***^	-0.170^***^	-0.140^***^

**P < 0.01,***P<0.001.

The bold font denotes statistically significant p-value.

### 3.6 The multiple regression analysis of online learning satisfaction

Regression analysis (**Table 5**) was performed based on the results of the preceding one-way analysis of variance and correlation analyses. Notable variations in OLS among genders, residence, and education levels were observed, necessitating the transformation of these variables into dummy variables for control purposes. Furthermore, Enter’s method was employed in the SPSS linear regression, where OLS was specified as the dependent variable and correlated and dummy variables were used as independent variables. Model I used health status and PR as independent variables, explaining a notable proportion of variance (*R* ^2 =^ 0.079, *P<*0.001); upon incorporating factors related to LS in Model II, the explanatory power increased significantly by 62.6% (*P<*0.001), with LS and PR emerging as significant predictors of OLS; in Model III (*β*=0.066,*P<*0.001; *β*=0.764, *P<*0.001), PC and NC were added to the equation, and the results showed that TCS did not significantly predict OLS (*β*=0.002, *P*>0.05; *β*=0.001, *P*>0.05).

**Table 5:** Multiple regression modelling of OLS.

Item			Model I			Model II			Model III		
			*β*	SE	*t*	*β*	SE	*t*	*β*	SE	*t*
constant			2.731	0.154	17.774^***^	0.764	0.108	7.071^***^	0.754	0.153	4.933^***^
independent variables		Health status	-0.108	0.029	-3.706^***^	-0.029	0.019	-1.546	-0.024	0.019	-1.253
		PR	0.291	0.037	7.940^***^	0.070	0.024	2.921^**^	0.066	0.025	2.674^**^
		LS				0.767	0.018	43.205^***^	0.764	0.018	42.696^***^
		PC							0.002	0.003	0.561
		NC							-0.001	0.002	-0.563
control variables	Residence	directly governed	0.027	0.132	0.206	-0.081	0.084	-0.97	-0.078	0.084	-0.931
		Provincial capital	-0.082	0.097	-0.839	-0.1	0.062	-1.617	-0.1	0.062	-1.615
		prefecture-level	-0.048	0.085	-0.568	-0.046	0.054	-0.849	-0.044	0.054	-0.814
		County-level	0.071	0.09	0.783	0.034	0.058	0.585	0.035	0.058	0.606
		Townships and Rural	0								
	Education	University	-0.149	0.075	-1.976	-0.048	0.048	-1.006	-0.049	0.048	-1.019
		High School	-0.157	0.097	-1.617	-0.018	0.062	-0.283	-0.016	0.062	-0.265
		Junior High School	0								
	Gender	Female	0.019	0.049	0.39	0.036	0.031	1.157	0.039	0.031	1.227
		Male	0								
R^2^			0.079			0.626			0.626		
Adjusted R^2^			0.072			0.623			0.623		
F			12.134			213.537			177.819		
*P*			<0.001			<0.001			<0.001		

All models control for demographic variables, ^**^P < 0.01,^***^P<0.001.

The bold font denotes statistically significant p-value.

### 3.7 Analysis of mediation effect

According to previous theories and literature reviews, TCS and PR played mediating roles between LS and OLS. In the present study, the bootstrapping method in SPSS PROCESS macro (3.5 version, Model 4) was used to investigate the moderated mediation effect of Parent-child Relationships and Trait Coping Styles on the relationship between Online Learning Satisfaction (26). The results show that there were no statistically significant mediating effects for PC(*CI*[-0.0013,0.0084]) and NC(*CI*[-0.0022,0.0124])between LS and OLS. However, PR played a significant positive mediating role in LS and OLS; the moderated mediating effect was 0.0132 (*P*<0.05), *CI* [0.0038,0.0244]; LS also had a significant positive direct effect on OLS,*β*=0.7714 (*P*<0.05), *CI* [0.7368,0.8060]; and a total effect on learning satisfaction,*β*=0.7847 (*P*<0.05), *CI* [0.7509,0.8184]. The mediating effect accounted for 1.682% of the total effect.

## 4 Discussion

This study examined factors influencing online learning satisfaction and has found that the parent-child relationship acted as a mediator between learning status and satisfaction, resulting in a positive impact. From the perspective of academic achievement, Wang (27) also found that the parent-child relationship between adolescents and their parents had a positive and direct effect on academic achievement, while there was a serial mediating effect of parental expectations and self-expectations; and in Liu’s study (28), parents’ self-efficacy was higher when they positively evaluated the online learning activities offered by the school, which in turn acted as a mediator to support their children’s online learning.

During the period of remote learning, a student’s learning status may directly impact their satisfaction with the learning experience. Simultaneously, it can indirectly affect their satisfaction by influencing their relationship with their parents. While there was a significant correlation between learning status and satisfaction, and learning status can be a useful predictor of satisfaction, the parent-child relationship may also serve as a mediator. This relationship potentially moderates the connection between the two variables, leading to uncertain learning satisfaction. The results suggest that enhancing parent-child relationships can serve as a favorable intervention for heightening academic contentment, particularly when students are in a despondent academic state. A negative parent-child relationship worsens the already low learning status, resulting in lower learning satisfaction. Conversely, a positive parent-child relationship contributes positively to excellent learning status and promotes continued learning satisfaction. This is consistent with the findings of some previous studies, such as the study by Núñez (29), which demonstrated that when parents are highly involved in their children’s homework, better academic performance is often observed and it may be related to the child’s higher self-efficacy and increased academic satisfaction and interest in learning;while low-quality parental involvement may result in parent-child conflict, negative parent-child relations, and reduced academic interest and learning satisfaction for the children (30). However, our study focused on the influence of the parent-child relationship on learning satisfaction as perceived by the children themselves. The similarity between this study’s findings and those of previous studies may be related to the emotional support received by the children, which affects their psychological motivation to learn.

The results of a study (31) on the effect of parent-child cohesion on academic achievement align with the hypotheses of this study. The findings suggest that parent-child cohesion, especially when mothers are more involved in their children’s schooling, can positively contribute to their children’s schooling at both emotional and psychological levels. We offer a new perspective on numerous research studies about the correlation between parent-child relationships and students’ academic performance. While previous studies (14) treated parent-child relationships as the independent variable affecting students’ academic outcomes, our findings suggest a mediated relationship. Although the mediating effect is minimal in the overall effect, it indicates an interaction between the two variables. The impact of parent-child relationships on students’ satisfaction with online learning appears to be delayed and obscured, indicating that students’ coping methods may have evolved due to these relationships over time. However, these changes may not be recognized by the students themselves, potentially leading to a disparity between their self-perceived and actual outcomes. Existing evidence (32) suggests that appropriate academic expectations or positive parental encouragement can mitigate and neutralize negative coping strategies in students.

Our survey demonstrated that Coping styles have a connection with satisfaction regarding online learning (*r*_PC_=0.110, *r*_NC_=-0.186, *P<*0.001), but the correlation is not linear (*P*_PC_>0.05, *P*_NC_ *>*0.05). A study conducted by Alconero-Camarero (33) revealed that there was no meaningful positive correlation between coping styles and academic satisfaction. Hence, it is not necessarily the case that students who employ maladaptive coping strategies experience lower academic satisfaction. In contrast, research on academic achievement among college students by Ortega-Maldonado and Salanova (34) suggested that positive adaptive coping styles are a significant predictor of learning satisfaction. The discrepancy between the results of these studies suggests that the prediction of learning satisfaction through coping styles may be influenced by various confounding factors. In our study, the parent-child relationship is considered as one such confounding factor.

Our aim is to assess students’ satisfaction with our studies, examining the parent-child relationship as a mediator. However, it is worth noting that both parents and their children are facing similar social situations during online learning and are challenged by their work and studies. In the future, we could endeavor to incorporate external factors into the model to examine whether variations exist in parents’ and children’s appraisals of the parent-child bond in identical environment, and the resultant impact on the work and education of both parties. In discussions about parenting influence, it is noteworthy that fathers and mothers have varying degrees of influence on their children’s schooling. Brent et al. illustrated that educational activities shared between fathers and children, such as reading or educational games, correlate more strongly with children’s cognitive outcomes than similar mother-child time (35). Additionally, fathers and mothers display different levels of adaptability and coping capacity when faced with similar environmental challenges, a variation that may be attributed to gender differences in coping strategies (36). As demonstrated by Spinelli (37), their study found that working mothers are more affected by lockdown policy and insufficient childcare, resulting in heterogeneity within the father-mother relationship with their children. This aspect warrants further exploration in future studies.

In summary, to enhance students’ online learning during the pandemic, attention should be given to on improving their coping strategies and strengthening the parent-child relationship. It is crucial to acknowledge that these two factors are interrelated. As the parent-child relationship becomes more cooperative and harmonious, children are better equipped to handle the challenges of school and life, even under high-pressure condition of an epidemic lockdown. Simultaneously, parents are more capable of managing their own work and offering enduring support to their children, particularly in the emotional realm. However, schools have an obligation to establish timely communication with parents, ensuring that they are well-informed with online learning programs and can support their children’s academic performance based on their individual characteristics. Teachers should also communicate with parents to offer personalized guidance, academic assistance, and, when necessary, specialized tutoring and psychological intervention.

### 4.1 Applicability and limitations

This study has broad applicability, revealing how strengthening parent-child relationships and cultivating positive coping strategies can enhance student satisfaction with online learning. As online learning platforms and distance education become increasingly prevalent globally, educators, school administrators, and policymakers can utilize these insights to devise and execute targeted interventions. These interventions could include the development of family involvement plans, enhancing communication and support between parents and children, and providing resources and training for students to develop positive coping skills–all of which are key factors in improving online learning satisfaction.

Nonetheless, this study is not without its limitations. First, due to the diversity and standardization difficulties of different course types and platforms, key covariates such as course characteristics, learning devices, and teaching methods were not included in the regression model, which may affect the accuracy of the results. Secondly, the cross-sectional study design limits causal inference. Single-time measurements may not fully reflect all aspects of concepts such as online learning satisfaction, learning status, parent-child relationships, etc., Future research should, therefore, contemplate employing more comprehensive measurement tools.

## 5 Conclusion

This study identifies learning status and parent-child relationships as significant determinants of satisfaction with online learning. The parent-child relationship is a key mediator, enhancing satisfaction levels, whereas learning status impacts satisfaction both directly by affecting the parent-child dynamic. These findings underscore the critical role of a supportive home environment in online learning success. Additionally, although coping styles are linked to satisfaction, their influence appears complex and non-linear, indicating a need for further research to clarify their impact. Overall, the study advocates that fostering positive family interactions and comprehending individual coping mechanisms may be crucial for improving online learning experiences.

## Data availability statement

The original contributions presented in the study are included in the article/**Supplementary Material**. Further inquiries can be directed to the corresponding author.

## Ethics statement

The studies involving humans were approved by The Ethics Committee of School of Medicine, Xiamen University. The studies were conducted in accordance with the local legislation and institutional requirements. Written informed consent for participation in this study was provided by the participants’ legal guardians/next of kin.

## Author contributions

LW: Conceptualization, Data curation, Formal analysis, Methodology, Writing – review & editing. WX: Investigation, Writing – original draft. LZ: Funding acquisition, Supervision, Validation, Writing – review & editing. XY: Writing – review & editing. XL: Writing – review & editing. CS: Funding acquisition, Supervision, Writing – review & editing.
