# Supplementary material for: An interaction study of online learning satisfaction with parent-child relationships and trait coping styles
Source: Front Psychiatry. 2024 Mar 1;15:1320886. doi: 10.3389/fpsyt.2024.1320886 (PMC10940432; doi:10.3389/fpsyt.2024.1320886)
Supplement: Supplementary file 1 [file DataSheet_1.doc]

Questionnaire

Tip：The purpose of this questionnaire is to investigate the factors related to online learning that affect the student population during the Covid-19 epidemic, as well as parent-child relationship status, and to observe students' coping styles in conjunction with the Trait Coping Styles Scale. The information collected will remain confidential. Thank you for your support and co-operation. By answering the questions, you are agreeing to take part in this study.

1. Gender

A、Male

B、Female

2、Level of school you are currently attending

A、Middle school

B、High school

C、College

3、Residence

A、Municipalities directly under the central government

B、Provincial capitals

C、Prefecture-level cities

D、County-level cities

E、Townships and villages

F、Unknown

1. Health status
2. Very good
3. Good

C、common

D、bad

E、Very bad

Learning

1. During the online learning period, the evaluation of your learning status was
2. Very dissatisfied
3. Dissatisfied
4. Average

D、Satisfied

E、Very satisfied

1. During the online learning period, your satisfaction with online learning was

A、Very dissatisfied

B、Dissatisfied

C、Average

D、Satisfied

E、Very satisfied

Parent-child relationship

How has your relationship with your parents changed during your online studies?

A、Very bad,

B、worse

C、almost the same

D、better

E、very good

Trait Coping Styles Scale

Q1、You'll be able to forget the unpleasantness as soon as possible.

1. Definitely not B、Shouldn't be C、Not sure D、Should be E、Definitely yes

Q2、You tend to get caught up in memories and fantasies of events that you can't get out of.

A、Definitely not B、Shouldn't be C、Not sure D、Should be E、Definitely yes

Q3、You act as if it never happened.

A、Definitely not B、Shouldn't be C、Not sure D、Should be E、Definitely yes

Q4、You tend to be angry with others and lose your temper often

A、Definitely not B、Shouldn't be C、Not sure D、Should be E、Definitely yes

Q5、You usually look on the bright side. Think positive.

A、Definitely not B、Shouldn't be C、Not sure D、Should be E、Definitely yes

Q6、Unpleasant events can easily cause mood swings

A、Definitely not B、Shouldn't be C、Not sure D、Should be E、Definitely yes

Q7、You like to keep your emotions to yourself, but you can't get them out of your head.

A、Definitely not B、Shouldn't be C、Not sure D、Should be E、Definitely yes

Q8、You usually compare yourself to similar people and think it's nothing.

A、Definitely not B、Shouldn't be C、Not sure D、Should be E、Definitely yes

Q9、You are quicker to turn negatives into positives, e.g. by participating in activities, etc.

A、Definitely not B、Shouldn't be C、Not sure D、Should be E、Definitely yes

Q10、It's easy to cry quietly when you're in trouble.

A、Definitely not B、Shouldn't be C、Not sure D、Should be E、Definitely yes

Q11、It's easy for a bystander to cheer you up again

A、Definitely not B、Shouldn't be C、Not sure D、Should be E、Definitely yes

Q12、If there is a conflict with someone, it is better to ignore them for a long time

A、Definitely not B、Shouldn't be C、Not sure D、Should be E、Definitely yes

Q13、Often indecisive and unable to think of solutions to major difficulties

A、Definitely not B、Shouldn't be C、Not sure D、Should be E、Definitely yes

Q14、Adapts quickly to difficulties and pain

A、Definitely not B、Shouldn't be C、Not sure D、Should be E、Definitely yes

Q15、Believe that difficulties and setbacks can train people

A、Definitely not B、Shouldn't be C、Not sure D、Should be E、Definitely yes

Q16、A long time to remember the unpleasant things that happened.

A、Definitely not B、Shouldn't be C、Not sure D、Should be E、Definitely yes

Q17、Often blame themselves for their incompetence and resent themselves when they encounter problems

A、Definitely not B、Shouldn't be C、Not sure D、Should be E、Definitely yes

Q18、Thinks there's no such thing as a big deal under the sun

A、Definitely not B、Shouldn't be C、Not sure D、Should be E、Definitely yes

Q19、I like to be alone when I'm in trouble.

A、Definitely not B、Shouldn't be C、Not sure D、Should be E、Definitely yes

Q20、 Usually defuses awkward situations with humor

A、Definitely not B、Shouldn't be C、Not sure D、Should be E、Definitely yes
